# Violence risk assessment in forensic psychology office: from childhood to elderly

**DOI:** 10.1080/07853890.2021.1896198

**Published:** 2021-09-28

**Authors:** Ricardo Ventura Baúto, Ana Filipa Carreiro, Margarida Pereira, Iris Almeida

**Affiliations:** a Laboratório de Ciências Forenses e Psicológicas Egas Moniz (LCFPEM), Centro de Investigação Interdisciplinar Egas Moniz (CiiEM), Egas Moniz Cooperativa de Ensino Superior, Caparica, Portugal; b Centro de Investigação Interdisciplinar Egas Moniz (CiiEM), Egas Moniz Cooperativa de Ensino Superior, Caparica, Portugal; c Laboratório de Psicologia Egas Moniz (LabPSI-EM), Centro de Investigação Interdisciplinar Egas Moniz (CiiEM), Egas Moniz Cooperativa de Ensino Superior, Caparica, Portugal; d Instituto Universitário Egas Moniz (IUEM), Egas Moniz Cooperativa de Ensino Superior, Caparica, Portugal

## Abstract

**Introduction:**

The purpose of this paper is to demonstrate the work developed by the Forensic Psychology Office (GPF) at Forensic Sciences and Psychology Laboratory located at the Egas Moniz Higher Education School. GPF’s main goals are performing forensic psychological assessments, especially violence risk assessments, as well as scientific research. The main purpose of violence risk assessment is the prevention and development of management strategies to minimise risk and try to identify factors that may contribute to the violent behaviour [1] supporting the criminal justice system in allocating more appropriate measures (e.g. sentence, intervention) [2]. GPF presents itself as the main response to cases with higher complexity and it provides guidance about the necessary measures to protect victims [3,4].

**Materials and methods:**

This is a quantitative study and the sample (*n* = 90) is derived from violence risk assessments of GPF (2016–2019). We evaluate 52 victims: 39 women/girls and 13 man/boys, aged between 5 and 95 years old (*M* = 33.04, *SD* = 21.82); and 38 defendants: 30 men and 8 women, aged between 23 and 82 years old (*M* = 44.64, *SD* = 14.75). Data was collected from lawsuits, semi-structured interviews of the victims and defendants, collateral information and clinical and forensic assessment tools. All participants signed an informed consent term, which contained the purpose of the assessment, the limits of the confidentiality, and also information about the ethics and technicians impartiality. All ethical principles have been taken due to the sensitive nature of the data involved and the respective informed consent.

**Results:**

In 90 criminal processes assessed, 66 cases was about reported situations of domestic violence. In these cases the relationship between victims and defendants was: 33 ex-partners; 12 ex-spouses; 10 ex-boyfriend/girlfriend; 6 married; 3 parents and 2 son/daughter. We assessed 11 child abuse cases (5 parents; 3 relatives; 2 son/daughter; 1 stepdaughter). We also evaluate 9 child sex abuse cases (2 son/daughter; 2 classmates; 2 stepdaughters; 2 relatives and 1 stranger). Finally, we evaluate 4 elderly abuse cases (2 relatives; 1 son/daughter and 1 parent). In the violence risk assessments, most of the cases presented high risk level (*n* = 33, 36.7%), followed by moderate risk (*n* = 23, 25.6%) and low risk (*n* = 11, 12.2). In defendant’s testimony credibility, 39.5% (*n* = 15) was undetermined, 34.2% probably not credible (*n* = 13), 7.9% (*n* = 3) probably credible and 2.6% (*n* = 1) did not collaborate in the assessment. In victim’s credibility of testimony, 73.1% (*n* = 38) was probably credible, 15.4% (*n* = 8) undetermined and 3.8% (*n* = 2) probably not credible.

**Discussion and conclusions:**

Higher and moderate risk are the most common levels in the Office assessed cases. These results demonstrate evidences of violence risk assessment importance in criminal justice system and an good practices example between Forensic Psychology and Law. Currently, through psychological assessment protocols defined for this purpose, the GPF has contributed to supporting the criminal justice system in allocating measures that are more appropriate to protect victims.
